# What the radiologist should know about artificial intelligence – an ESR white paper

**DOI:** 10.1186/s13244-019-0738-2

**Published:** 2019-04-04

**Authors:** European Society of Radiology (ESR), Emanuele Neri, Nandita de Souza, Adrian Brady, Angel Alberich Bayarri, Christoph D. Becker, Francesca Coppola, Jacob Visser

**Affiliations:** European Society of Radiology, Am Gestade 1, 1010 Vienna, Austria

**Keywords:** Artificial intelligence, Imaging informatics, Radiomics, Ethical issues, Computer applications

## Abstract

This paper aims to provide a review of the basis for application of AI in radiology, to discuss the immediate ethical and professional impact in radiology, and to consider possible future evolution.

Even if AI does add significant value to image interpretation, there are implications outside the traditional radiology activities of lesion detection and characterisation. In radiomics, AI can foster the analysis of the features and help in the correlation with other omics data. Imaging biobanks would become a necessary infrastructure to organise and share the image data from which AI models can be trained. AI can be used as an optimising tool to assist the technologist and radiologist in choosing a personalised patient’s protocol, tracking the patient’s dose parameters, providing an estimate of the radiation risks. AI can also aid the reporting workflow and help the linking between words, images, and quantitative data. Finally, AI coupled with CDS can improve the decision process and thereby optimise clinical and radiological workflow.

## Key points

- Outside the traditional radiology activities of image interpretation, AI is estimated to impact on radiomics, imaging biobanks, clinical decision support systems, structured reporting, and workflow.
- The key factor of AI performance is training with big and high-quality data to avoid overfitting and underfitting
- The three laws of robotics could be applied to radiology where the “robot” is the “AI medical imaging software.”
- If AI is used in clinical practice, the main medico-legal issue that then arises is “who is responsible for the diagnosis.”

## Introduction

Artificial Intelligence (AI) is one of the fastest-growing areas of informatics and computing with great relevance to radiology. A recent PubMed search for the term “Artificial Intelligence” returned 82,066 publications; when combined with “Radiology,” 5,405 articles were found. Most of these papers have been published since 2005. Practicing radiologists, trainees, and potential future radiologists need to understand the implications of AI for the specialty, what it means, how it can contribute to the radiological profession, and how may change it in the future. The European Society of Radiology (ESR) is aware of the impact that AI is having on the field of Radiology, from technical-scientific, ethical-professional, and economic perspectives. Much fear has been generated among radiologists by the statements in public media from researchers engaged in AI development, predicting the imminent extinction of our specialty. For example, Andrew Ng (Stanford) stated that “[a] highly-trained and specialised radiologist may now be in greater danger of being replaced by a machine than his own executive assistant” [1], whereas Geoffrey Hinton (Toronto) said “[i]f you work as a radiologist, you’re like the coyote that’s already over the edge of the cliff, but hasn’t yet looked down so doesn’t realise there’s no ground underneath him. People should stop training radiologists now. It’s just completely obvious that within 5 years, deep learning is going to do better than radiologists […] We’ve got plenty of radiologists already [.]” [2].

As one of the responsibilities of the ESR eHealth and Informatics subcommittee, this paper aims to provide a review of the basis for application of AI in radiology, to discuss the immediate ethical and professional impact of AI in radiology, and to consider possible future evolution of such technology within diagnostic imaging.

## Definitions

Artificial Intelligence (AI) represents the capacity of machines to mimic the cognitive functions of humans (in this context, learning and problem solving). AI can be subdivided into artificial narrow intelligence, where a computer can perform a very specific task as well as or better than humans (e.g., IBM’s Watson computer which beat two Jeopardy champions in 2011), and artificial general intelligence, where a computer goes beyond specific tasks to perform higher-order syntheses, emulating human thought processes [3]. In 1950, British computer scientist Alan Turing enunciated the basis of the Turing test: a computer passes the test if a human interrogator, after posing a number of written questions, cannot tell whether the written responses come from a person or a computer [4, 5]. A refinement of this is the so-called Smith test: data is provided to a computer to analyse in any way it wants; the computer then reports the statistical relationships it thinks may be useful for making predictions. The computer passes the Smith test if a human panel concurs that the relationships selected by the computer make sense [6].

AI can be understood as a set of tools and programs that make software “smarter” to the extent that an outside observer thinks the output is generated by a human. It operates similarly to the way a normal human brain functions during regular tasks like common-sense reasoning, forming an opinion, or social behaviour [7].

The term “artificial intelligence” was first used in 1956 at the summer workshop at Dartmouth College in Hanover, New Hampshire, organised by John McCarthy, an American computer scientist, pioneer, and inventor [8].

The term machine learning (and its subcategories) implies the situation in which an *agent* (anything that can be viewed as perceiving its environment through *sensors* and acting upon that environment through *actuators*) is learning if it improves its performance on future tasks after making observations about the world [9]. Machine learning is a term introduced by Arthur Samuel in 1959 to define a field of AI in which computers learn automatically from data accumulation; it has been extensively applied to big data analysis. Machine learning algorithms evolve with increasing exposure to data; they are not based exclusively on rules, but improve with experience, learning to give specific answers by evaluating large amounts of data [10].

The learning can be unsupervised, reinforced, supervised, and semi-supervised. In *unsupervised learning*, the agent learns patterns in the input even though no explicit feedback is supplied. In *reinforcement learning*, the agent learns from a series of reinforcements—rewards or punishments. *Supervised learning* provides the agent with a teacher’s output that the agent then learns. In *semi-supervised learning*, fewer teacher’s outputs are given to the agent. In this context, the concept of “ground truth,” which means checking the results of machine learning for accuracy against the real world, is fundamental for validating AI performance. In a radiology context, this might mean confirming diagnoses suggested by AI by comparison to pathological or surgical diagnoses; ground truth is the data assumed to be true [11]. Machine learning has been likened to training a dog: “reinforcing good behaviour, ignoring bad, and giving her enough practice to work out what to do for herself” [12].

*Deep learning* is a subset of machine learning and is the basis of most AI tools for image interpretation. Deep learning means that the computer has multiple layers of algorithms interconnected and stratified into hierarchies of importance (like more or less meaningful data). These layers accumulate data from inputs and provide an output that can change step by step once the AI system learns new features from the data. Such multi-layered algorithms form large artificial neural networks [9].

*Artificial Neural Networks* are composed of nodes or *units* (thousands to millions) connected by links. A link propagates *activation* from one unit to another, and each link activation is weighted by a numeric value which determines the strength of the connection. The activation function can be based on a threshold of activation (the strength), and the unit that receives the activation is called a *perceptron*. Perceptrons are connected by links and create a network; the network can be *feed-forward* (where connections are only in one direction) or it could be a *recurrent network*, which feeds its outputs back into its own inputs (a loop). Feed-forward networks are usually arranged in *layers***.** The goal is that the answer for each examination should match the examination’s labels. Mathematically, the algorithm is designed to maximise the number of right answers as the inputs are processed through its layers [9].

Artificial neural networks must be “trained” using training data sets from which the network “learns.” In radiology, these usually consist (at least initially) of hand-labeled image data sets used by the algorithm to improve its fit to match the ground truth. Once a network has been trained using a training data-set, it would then be tested using a different set of data (validation data sets), designed to evaluate the fit of the model to new data. In this step, it is common to observe “*overfitting*” of the model. Yamashita et al. describe overfitting as the situation *“*where a model learns statistical regularities specific to the training set, i.e., ends up memorising the irrelevant noise instead of learning the signal, and, therefore, performs less well on a subsequent new dataset.” [13]. The consequence of overfitting is that the network will not be generalisable to never-seen-before data and will be a source of more interpretation errors than those which occurred in the training data set.

The best solution for reducing overfitting is to obtain more training data. Multiple rounds of training and testing on different datasets may be performed, gradually improving network performance, and permitting assessment of the accuracy and generalisability of the algorithm, before the algorithm is released for general use. Another solution is the so-called “data augmentation” which means modifying the training data by adding some variability so that the model will not see the exact same inputs from the training data set during the training iterations. A simple example: if the network is being trained to recognise a cystic lesion on ultrasound, on the training data set the lesion could be perfectly cystic, but in a further iteration of training, some internal hyperechoic spots caused by artifacts could be added in order to train or fine-tune the network to recognise a “non-perfect cyst” in the validation data sets [13].

As a general rule, the “deeper” the network (more layers) and the more rounds of training, the better the performance of the network.

## Use cases

The term “use case” describes a specific clinical application of AI in radiology. Use cases can be considered as precise scenarios within the radiology service chain where automation could add significant value and establish standards.

Computer-aided detection (CAD) represents the earliest clinical applications of basic AI in radiology. CAD system has been progressively implemented in radiological practice in the last two decades in the detection of lung, colon, breast, and prostate cancer, but the beginning of research in CAD, according to Kunio Doi [14], a scientist and pioneer in CAD research, can be attributed to articles published between 1963 and 1973 [13, 15–19]. Multiple CAD applications have been reported since then, and CAD has become common in clinical practice, with the main application to the detection of lung, colon, and breast cancers [20–22].

The main difference between CAD and “true” AI is that CAD only makes diagnoses for which it has been specifically trained and bases its performance on a training dataset and a rigid scheme of recognition that can only be improved if more datasets are given to the CAD algorithm. True AI is characterised by the process of autonomous learning, without explicit programming of each step, based on a network of algorithms and connections, similar to what humans do.

In the last decade, there has been an explosion in studies employing artificial intelligence for image interpretation that embrace disease detection and classification, organ and lesion segmentation (determining the boundaries of an organ or lesion), and assessment of response to treatment. However, it is difficult to discriminate papers related to the use of CAD and those reporting the pure application of machine or deep learning, since both terms are included in the wider term “artificial intelligence.” Some of many recent applications of AI include the RSNA paediatric bone age machine learning challenge on plain radiographs [23], breast cancer detection in mammography and MRI [24–29], chest radiograph interpretation [30–33], liver lesion characterisation on ultrasound and CT [34–36], brain tumour [37, 38], and prostate cancer detection [39, 40].

A step beyond disease detection is disease classification into low or high risk, with good or poor prognosis. Much of the work in this field has been in brain imaging, in both benign and malignant disease. There has been considerable effort to develop AI classifiers in paediatrics, where brain mapping and functional connectivity can be linked to neurodevelopmental outcome. In a study evaluating resting state-functional MRI data from 50 preterm-born infants, binary support vector machines distinguished them from term infants with 84% accuracy (p < 0.0001), based primarily on inter- and intra-hemispheric connections throughout the brain [41]. In multiple sclerosis, AI has been used to evaluate the performance of combinations of MRI sequences to optimise brain lesion detection [42]. Classification of glioma grade based on MR images has been attempted with some success [43].

Automated segmentation is crucial as an AI application for reducing the burden on radiology workflow of the need to perform segmentation manually. It also provides vital information on the functional performance of tissues and organs, disease extent, and burden. Avendi et al. developed a deep learning and deformable model for left ventricular (LV) segmentation from cardiac MRI datasets, to obtain an automated calculation of clinical indices such as ventricular volume and ejection fraction [44]. Multiple studies have been published about abdominal (liver, pancreas, vessels) and pelvic (prostate) organ segmentation using a deep learning approach [45–51].

A similar approach has been applied to segmenting brain metastases on contrast-enhanced T1W MR for planning for stereotactic radiosurgery [52].

## Other applications of AI in Radiology

Outside the traditional radiology activities of lesion detection and characterisation, and assessment of response to treatment, AI is likely to impact other areas of radiologists’ and other healthcare professionals’ work. Examples include:

- Radiomics: extraction of features from diagnostic images, the final product of which is a quantitative feature/parameter, measurable and mineable from images. A Radiomics analysis can extract over 400 features from a region of interest in a CT, MRI, or PET study, and correlate these features with each other and other data, far beyond the capability of the human eye or brain to appreciate. Such features may be used to predict prognosis and response to treatment [53, 54]. AI can support analysis of radiomics features and help in the correlation between radiomics and other data (proteomics, genomics, liquid biopsy, etc.) by building patients’ signatures [55].
- Imaging biobanks: the constantly enlarging memory capacity of computers permits storage of large amounts of data. In radiology, the need to store native images and big data derived from quantitative imaging represents the main cause of PACS overload. Quantitative imaging can produce imaging biomarkers that can be stored and organised in large imaging biobanks (potentially using data from many institutions and locations), available to be processed, analysed, and used to predict the risk of disease in large population studies and treatment response [56, 57]. Large biobanks also have the potential to become the repository of digital patients (Avatars or Digital Twins of humans) that can be used by AI to perform simulations of disease development and progression. Moreover, imaging biobanks would become a necessary infrastructure to organise and share the image data from which AI models can be trained.
- Dose optimisation: The ESR EuroSafe Imaging initiative is designed to support and strengthen medical radiation protection across Europe following a holistic, inclusive approach (www.eurosafeimaging.org). EuroSafe Imaging promotes the adoption of clinical diagnostic reference levels in CT that should be customised based on the appropriateness criteria and on patient characteristics (BMI, circulation time, etc.). The choice of protocol, however, is subject to variability since it is frequently operator-dependent, and consequently, the radiation dose and the quality of the exam are subject to variability at both intra- and inter-institutional levels. In this setting, AI can be an optimising tool for assisting the technologist and radiologist in choosing a personalised patient’s protocol, in tracking the patient’s dose parameters, and in providing an estimate of the radiation risks associated with cumulative dose and the patient’s susceptibility (age and other clinical parameters).
- Structured reporting: AI can aid the reporting workflow and help the linking between words, images, and quantitative data, and finally suggest the most probable diagnosis. The structured reporting initiative of RSNA, in which ESR is a partner, is proposing the adoption of “common data elements” (CDEs) that define the attributes and allowable values of a unit of information so that information can be collected and stored uniformly across institutions and studies [58]. CDEs are defined in a data dictionary and make biomedical data interoperable for a variety of applications, including clinical radiology reports, computer-assisted reporting systems, structured image annotations, case report forms for clinical research, and radiology case collections (“teaching files”) [59]. CDEs can be the vocabulary of AI to build a patient’s specific structured report.
- AI tools can also impact the daily workflow by filtering exam priority based on appropriateness criteria [60]. ESR has implemented the “ESR iGuide,” a clinical decision support system (CDS) that assists referring physicians to choose the most appropriate imaging procedure based on the level of evidence for appropriateness, and the level of emergency [61]. AI coupled with CDS can improve the decision process and thereby optimise clinical and radiological workflow.

### Barriers to AI in radiology & challenges

#### Data-sets & training

The availability of large amounts (big data) of medical images in the imaging domain (from PACS systems) offers great potential for AI training, but such data need a so-called “curation” process in which the data are stratified by patient cohorts, segmented to extract the region of interest for AI interpretation, filtered to assess the quality of acquisition and reconstructions, etc. [62].

However, a data-set annotation is very time- and labor-intensive, and the validation of ground truth diagnosis must be very robust. Rare findings are a potential weakness; if a condition or a finding is very rare, it’s difficult to obtain enough examples to train an algorithm to identify it with confidence. Also, variation in findings can lead to inadvertent *overfitting*, where random noise is interpreted by the algorithm as an abnormality. Conversely, if the training data set used to train an algorithm contains inherent biases (e.g., ethnic-, age- or gender-based), the algorithm may *underfit* findings from data derived from a different patient population [60].

#### Regulation issues

Humans would like Isaac Asimov’s fictional three laws of robotics [63] to be applied to AI in radiology, where the “robot” is the “AI medical imaging software”:

1. “A robot may not injure a human being or, through inaction, allow a human being to come to harm.”
2. “A robot must obey the orders given it by human beings except where such orders would conflict with the First Law.”
3. “A robot must protect its own existence as long as such protection does not conflict with the First or Second Laws.”

The first law implies that *AI tools should achieve the best possible diagnosis to improve patient’s healthcare*; however, AI failure or inaction could result in harm to the patient.

The second law suggests that *AI must be properly trained* and that the learning process of any AI software must be supervised by a radiologist, to ensure appropriate and clinically-applicable outputs.

The third law may be an issue when considering the inevitable eventual obsolescence of any AI software. Imaging is evolving so rapidly that the training of a software, with specific imaging data, can be insufficient once a new development of the modality (CT, MRI, US, NM) or new modalities are introduced into clinical practice [64, 65].

Unfortunately, Asimov’s laws are fictional, and no regulatory body has the ultimate power or control to ensure they (or common sense analogs) are embedded in any specific AI product. At present, we rely on the ethical behaviour of software developers and their collaborators to ensure that AI products behave and perform according to appropriate standards. When AI software is applied to human clinical care, it should be subject to the same form and standard of regulation as any other medical device or product, as provided for by the FDA (in the US) or the EU Medical Device Regulation 2017 [66]. Only by applying the same stringent standards of accountability, effectiveness, and validation of clinical usefulness that would be applied to a new drug or device can we ensure patient safety when AI is applied to patient diagnosis.

#### Medico-legal responsibility

CAD, as applied to CT colonography, lung cancer, and breast cancer detection, can be used as a first reader (not recommended by the literature), second reader (mostly recommended) or as a concurrent reader (few recommendations), but never as an independent reader of studies. How will AI be used? Probably the CAD models will be re-proposed but, as stated by Kholi et al., “[t]he power and promise of the AI approach over traditional CAD is that useful features can exist that are not currently known or are beyond the limit of human detection.” It means that AI may bring new features that radiologists cannot detect or quantify [67]. A similar example is radiomics, where a texture analysis can generate hundreds of features that a human being cannot generate and interpret.

The medico-legal issue that then arises is the question of “who is responsible for the diagnosis,” especially if it is wrong. Whether data scientists or manufacturers involved in development, marketing, and installation of AI systems will carry the ultimate legal responsibility for adverse outcomes arising from AI algorithm use is a difficult legal question; if doctors are no longer the primary agents of interpretation of radiological studies, will they still be held accountable? If radiologists monitor AI system outputs and still have a role in validating AI interpretations, do they still carry the ultimate responsibility, even though they do not understand, and cannot interrogate the precise means by which a diagnosis was determined? This “black box” element of AI poses many challenges, not least to the basic human need to understand how and why important decisions were made. How many of us would be content if we were told by our doctor: “I don’t know why you’re ill, but my computer says ‘take these pills’,” or “I don’t know why you’re ill, but my computer recommends surgery”? [6].

#### Need for access to large volumes of data

Access to large volumes of data is necessary to develop and train AI algorithms; this can be one of the major factors limiting algorithm development. Software developers use a variety of methods to obtain training datasets; some liaise directly with patients, others with institutions or academic databases. Fundamentally, each patient whose data is used by a third party should provide consent for that use, and that consent may need to be obtained afresh if the data is re-used in a different context (e.g., to train an updated software version). Moreover, ownership of imaging datasets varies from one jurisdiction to another. In many countries, the ultimate ownership of such personal data resides with the patient, although the data may be stored, with consent, in a hospital or imaging centre repository.

Anonymisation of data should be guaranteed; this involves more than de-identification and should ensure no capability to re-identify the patient through DICOM tags, facial recognition software, etc. [68].

Furthermore, if patient data are used to build AI products which go on to generate profit, consideration needs to be given to the issue of intellectual property rights. Do the involved patients and the collecting organisations have a right to share in the profits that derive from their data? [68].

#### Data mining & radiomics

One of the many exciting prospects for AI incorporation into radiology practice is the potential to extract data from large numbers of studies, unconstrained by location, and use that data to identify imaging markers that may predict outcome or response to treatment – Radiomics. Neural network analysis of large datasets can isolate important relationships that could never be perceived by visual interpretation of studies alone, and which may prove important in future personalised healthcare. However, given that these relationships are the products of mathematical algorithms, they may not always make sense or be relevant. The Texas sharpshooter fallacy is a term applied to the reasoning that may apply: a sharpshooter covers a wall with targets, fires his gun, and displays whatever target he hits (or draws a target around his bullet hole, having fired at a blank wall) to display his prowess, ignoring all the targets missed. This form of analysis has been described as “data first, theory later.” In the words of the 1991 Nobel Laureate in Economics, Ronald Coase, “if you torture the data long enough, it will confess.”

This is not intended to suggest that all data mining or radiomics falls into this fallacy; far from it. However, some data mining outputs will be irrelevant or irrational.

#### Other ethical issues

The ethical issues arising from AI use in radiology will be considered in greater detail in a multi-society (including the ESR) paper currently in preparation and will not be explored in depth here. Some of the points considered above encompass ethical elements. Fundamentally, it must be remembered that AI tools are mathematical constructs, designed to optimise a mathematical function. Intrinsically, they are amoral. The challenge for humans is to anticipate how AI systems can go wrong or could be abused and to build in protections against this happening [68].

## AI, radiology training and future directions

Radiologists’ skills are based on many years of training during which the trainee is taught to interpret large numbers of examinations based on a combined process of reading coupled with the knowledge of clinical information. Interpretation skills strongly depend on the number of exams interpreted and the accuracy of the visual image analysis. AI can perform image reading by exploiting deep learning tools and is able not only to extract visual information but also quantitative information, such as Radiomic signatures or other imaging biomarkers, which would not be identified by the human brain. AI is going to become part of our image viewing and analysis toolset. When software becomes part of the process of interpretation, trainees may not make enough direct (“unaided”) interpretations during their training years and therefore may not acquire adequate interpretation skills. The other face of this coin is that trainees will be helped by AI to perform better interpretations; nonetheless, a strong dependence of future radiologists on aid from AI software is a risk, with potentially deleterious consequences.

The implementation of AI in radiology requires that trainees learn how to best integrate AI in radiological practice, and therefore a specific AI and informatics module should be included in the future radiology training curricula.

AI involvement in our professional lives is inevitable. We need to work with software developers and computer engineers to assist the process of integration of AI tools into our workflows (PACS/RIS systems, task automation, etc.), always protecting the interests of patients primarily.

A vast amount of AI research is ongoing; image interpretation is an attractive target for researchers, given that the tasks involved (at least in part) involve analysis of large amounts of data to produce an output. Radiologists cannot, and should not, wish this research away, but rather embrace it and integrate it as much as possible into the daily work, guiding AI research directions to ensure the maximum clinical benefit to patients from new developments.

Another task that must be taken on is leadership in educating policymakers and payers about radiology, AI, their integration, and the associated pitfalls. In any rapidly developing industry, there is initial excitement, often followed by disappointment when early promises are unfulfilled [69].

The hype around new technology, often commercially-driven, may promise more than it can deliver, and tends to underplay difficulties. It is the responsibility of clinical radiologists to educate themselves about AI in radiology, and in turn to educate those who manage and fund our hospitals and healthcare systems, to maintain an appropriate and safe balance protecting patients while implementing the best of new developments.

### Will radiologists be replaced by AI?

The simple answer is: NO. However, radiologists’ working lives will undoubtedly change in this era of artificial intelligence. Many of the single routine tasks in the radiology workflow will be performed faster and better by AI algorithms, but the role of the radiologist is a complex one, focused on solving complex clinical problems [68]. The real challenge is not to oppose the incorporation of AI into the professional lives (a futile effort) but to embrace the inevitable change of radiological practice, incorporating AI in the radiological workflow [12]. The most likely danger is that “[w]e’ll do what computers tell us to do, because we’re awestruck by them and trust them to make important […] decisions” [6]. Radiologists can avoid this by educating themselves and future colleagues about AI, collaborating with researchers to ensure it is deployed in a useful, safe, and meaningful way, and ensuring that its use is always directed primarily towards the patient benefit. In this way, AI can enhance radiology, and allow radiologists to continually improve their relevance and value [70, 71].

## Abbreviations

AI: Artificial Intelligence
CAD: Computer-aided detection
CDE: Common Data Elements
CDS: Clinical Decision Support System
ESR: European Society of Radiology
